# Comparison of urinary and sexual patient-reported outcomes between open radical prostatectomy and robot-assisted radical prostatectomy: a propensity score matched, population-based study in Victoria

**DOI:** 10.1186/s12894-022-00966-0

**Published:** 2022-02-07

**Authors:** Michael Rechtman, Andrew Forbes, Jeremy L. Millar, Melanie Evans, Lachlan Dodds, Declan G. Murphy, Sue M. Evans

**Affiliations:** 1 School of Public Health and Preventive Medicine, Monash University, 533 St Kilda Road, Melbourne, VIC 3004 Australia; 2 Radiation Oncology, Alfred Health, South Block Ground, 55 Commercial Road, Melbourne, VIC 3004 Australia; 3 Department of Urology, Ballarat Health Services, Ballarat, Australia; 4 St. John of God Hospital Ballarat, Ballarat, Australia; 5 Epworth Prostate Centre, Epworth Healthcare, Richmond, VIC Australia; 6 Sir Peter MacCallum Department of Oncology, University of Melbourne, Parkville, VIC 3052 Australia; 7 Victorian Cancer Registry, 615 St Kilda Rd, Melbourne, VIC 3004 Australia

**Keywords:** Radical prostatectomy (RP), Open radical prostatectomy (ORP), Robot-assisted radical prostatectomy (RARP), Patient-reported outcomes (PRO), Expanded prostate cancer index composite (EPIC) questionnaire

## Abstract

**Background:**

Robot-assisted radical prostatectomy (RARP) rates have been increasing worldwide despite a lack of evidence of superior patient-reported outcomes (PROs) compared to open radical prostatectomy (ORP).

**Methods:**

This retrospective study included men who contributed data to the Prostate Cancer Outcomes Registry-Victoria (PCOR-Vic), underwent ORP or RARP between January 2014 and May 2018, and completed the EPIC-26 questionnaire 12 months post-surgery. Urinary and sexual bother items, the urinary incontinence domain score, the urinary irritative/obstructive domain score, the sexual domain score and the pad usage item from the EPIC-26 questionnaire were compared between the two cohorts. Unmatched and propensity score matched cohorts were used to determine if there were differences in urinary and sexual PROs between ORP and RARP after accounting for the patient case-mix and surgeon characteristics.

**Results:**

Of 3826 patients undergoing radical prostatectomy (RP), 1047 received ORP and 2779 received RARP. Propensity score matching reduced the magnitude of the observed differences in four out of six outcomes (urinary bother, urinary incontinence domain, pad usage and sexual domain). Using a propensity score matched cohort, there were no statistically significant differences for RARP patients, compared to ORP patients, in terms of urinary bother (Rd = 0.47%, *P* = 0.707), urinary incontinence domain scores (Coeff = − 0.84, *P* = 0.506), urinary irritative/obstructive domain scores (Coeff = 1.03, *P* = 0.105), pad usage (Rd = − 0.75%, *P* = 0.771) and sexual bother (Rd = − 0.89%, *P* = 0.731). RARP patients had slightly higher sexual domain scores (Coeff = 3.65, *P* = 0.005).

**Conclusion:**

There were no differences in urinary PROs between ORP and RARP when assessed 12 months post-surgery. The sexual domain slightly favoured RARP, however this was not deemed clinically significant.

**Supplementary Information:**

The online version contains supplementary material available at 10.1186/s12894-022-00966-0.

## Background

Radical prostatectomy (RP) is the most commonly used treatment for patients with localised prostate cancer and life expectancy greater than 10 years [1]. Open radical prostatectomy (ORP) and robot-assisted radical prostatectomy (RARP) are the two main RP approaches performed in Victoria [2]. The increased availability of robotic surgery has led to the use of a robotic approach in the majority of RPs in Victoria [3].

The American Urological Association and the European Association of Urology guidelines for localised prostate cancer state that there is no evidence of different urinary or sexual functional outcomes between ORP and RARP, however the evidence level is rated low in both [4, 5]. The largest randomised controlled trial (RCT) comparing ORP and RARP found no difference in oncological and urinary and sexual functional outcomes at 12- and 24-months following surgery [6, 7]. However, minimally invasive surgeries such as RARP have gained popularity globally due to the potential for reduced morbidity [8]. This may be due to improved perioperative outcomes, such as decreased blood loss, improved short-term postoperative outcomes, such as reduced postoperative pain and length of hospital stay [6], and clinician and patient preference [8]. Marketing campaigns, hospital and urologist competition, and centralised health systems may have also contributed to the perception that RARP is superior [9, 10]. Furthermore, patient and surgeon characteristics may differ significantly between ORP and RARP cohorts [11]. Due to conflicting evidence and its increasing adoption [12], ongoing comparison of outcomes is warranted that is focussed on issues that impact quality of life. For this reason, observational studies and prostate cancer clinical registries have grown in number [13]. Population-based clinical registries allow stronger inferences to be made about the population compared to single- or multi-centre studies due to volume-outcome relationships [14].

This paper used a population-based clinical registry to compare urinary and sexual PROs from the EPIC-26 questionnaire of men undergoing ORP and RARP at one-year following surgery.

## Methods

### PCOR-Vic

Data for this study were obtained from the PCOR-Vic, which collects clinical and patient-reported data on men diagnosed with prostate cancer from contributing institutions in Victoria [15]. Recruitment and data collection methods of the PCOR-Vic have previously been described [15]. At approximately 12 months post-diagnosis for men on active surveillance or watchful waiting, and 12 months post initial active treatment, participants are invited to complete the EPIC-26 quality of life questionnaire [16] via telephone, email or paper form [15].

### Data collection

PCOR-Vic provided a dataset with a range of demographic, diagnostic and follow-up variables. Socioeconomic status was calculated using the patient’s postcode to determine the Australian Bureau of Statistics index of relative socio-economic advantage and disadvantage (IRSAD) for the 2016 year [17]. Year of attaining surgical specialisation was collected from the Australian Health Practitioner Regulation Agency website or directly from surgeons.

### Patients

Patients were included if they attended a participating institution of the PCOR-Vic, did not opt out of the registry, received an ORP or RARP between January 2014 and May 2018, and completed at minimum the urinary and sexual bother items of the EPIC-26 questionnaire between 0.7 to 1.3 years following RP.

### Outcomes

Urinary outcomes were the EPIC-26 urinary bother item (dichotomised into moderate/big bother, and small/very small/no bother, consistent with cut-off points reported elsewhere) [14, 18], urinary incontinence and urinary irritative/obstructive domain scores, and pad usage (dichotomised into ‘no pads per day’ and ‘≥ 1 pad per day’) [19]. Sexual outcomes were the EPIC-26 sexual bother item (dichotomised into moderate/big bother, and small/very small/no bother) and sexual domain scores [19].

### Statistical analysis

Descriptive analyses included medians and interquartile ranges for continuous data. Associations were determined using independent t-tests for continuous data and chi-squared tests for categorical data. A two-tailed 5% significance level was used throughout.

Propensity score matching was used to assess differences in urinary and sexual outcomes between ORP and RARP after accounting for the differences in patient and surgeon characteristics. Detailed steps involved in propensity score matching are described elsewhere [20, 21]. The propensity score refers to the estimated probability of receiving one of the treatment options.  It was defined as the probability of receiving RARP, as opposed to ORP. Propensity scores were calculated for each patient using a logistic regression model based on the preoperative factors of age at surgery, PSA at surgery, surgeon’s years since specialisation, National Comprehensive Cancer Network (NCCN) risk category, hospital location (metropolitan vs. regional), hospital type (public vs. private), IRSAD quintile and year of surgery.

The propensity score matching estimator used was a nearest neighbour 1:1 matching model, with replacement and no calipers. Therefore each patient in both treatment groups was matched to a patient in the other treatment group based on propensity scores. This model was used to define similarity in order to find the closest matches across the population. The matching estimator imputed the missing potential outcome for each individual (i.e. the outcome if they had received the other treatment option). Each potential outcome became an observation in the data.

In this manner, two groups of patients were formed that were similar on their propensity scores. After matching, the covariates used in calculating the propensity scores were checked for balance across the two treatment groups using a maximum standardised difference of 10%, as previously recommended [20, 22].

The average treatment effect for each outcome was estimated before and after propensity score matching. These were expressed as risk differences (RARP minus ORP) for binary variables and as mean differences (RARP minus ORP) for continuous variables.

All analyses were conducted using STATA version 15.1, with *p* values ≤ 0.05 considered statistically significant. The matched analyses used the *teffects* command. Ethics approval was obtained from the Monash University Human Research Ethics Committee (ID: 19196).

## Results

Of the 3826 patients included in this study, 1047 (27%) underwent ORP and 2779 (73%) underwent RARP.

Differing baseline characteristics included that RARP patients were more likely to have low or intermediate NCCN disease risk (78.3% vs. 72.0%, *p* < 0.001) and reside in postcodes in the top quintile of the IRSAD (43.9% vs. 34.3%, *p* < 0.001), compared to ORP patients (Table 1). RARP patients were also more likely to have surgery at a private institution (85.3% vs. 60.6%, *p* < 0.001) and have surgery at a metropolitan institution (93.5% vs. 71.5%, *p* < 0.001), compared to ORP patients.

**Table 1:** Baseline patient characteristics at diagnosis and surgery

Factor	ORP	RARP	*P* value
N	1047 (27.4%)	2779 (72.6%)	
*Year of diagnosis*			
2009–2012	3 (0.3%)	29 (1.0%)	0.05
2013	31 (3.0%)	64 (2.3%)	
2014	184 (17.6%)	507 (18.2%)	
2015	226 (21.6%)	671 (24.1%)	
2016	308 (29.4%)	750 (27.0%)	
2017	279 (26.6%)	731 (26.3%)	
2018	16 (1.5%)	27 (1.0%)	
*Method of diagnosis*			
TRUS prostate biopsy	516 (49.3%)	1160 (41.7%)	< 0.001
TURP	16 (1.5%)	35 (1.3%)	
Transperineal prostate biopsy	511 (48.8%)	1575 (56.7%)	
Other	4 (0.4%)	9 (0.3%)	
*PSA at diagnosis (ng/mL)*			
< 4.8	212 (20.2%)	768 (27.6%)	< 0.001
4.81–6.4	238 (22.7%)	690 (24.8%)	
6.41–9	288 (27.5%)	652 (23.5%)	
> 9	280 (26.7%)	593 (21.3%)	
Missing	29 (2.8%)	76 (2.7%)	
*ISUP Grade Group at diagnosis*			
1	159 (15.2%)	417 (15.0%)	< 0.001
2	412 (39.4%)	1301 (46.8%)	
3	237 (22.6%)	578 (20.8%)	
4	130 (12.4%)	272 (9.8%)	
5	104 (9.9%)	181 (6.5%)	
Missing	5 (0.5%)	30 (1.1%)	
*NCCN risk category*			
Low risk	89 (8.5%)	254 (9.1%)	< 0.001
Intermediate risk	660 (63.0%)	1897 (68.3%)	
High risk	256 (24.5%)	550 (19.8%)	
Metastatic	36 (3.4%)	47 (1.7%)	
Missing	6 (0.6%)	31 (1.1%)	
*Diagnosing institution type*			
Public	345 (33.0%)	415 (14.9%)	< 0.001
Private	667 (63.7%)	2180 (78.4%)	
Missing	35 (3.3%)	184 (6.6%)	
*Diagnosing institution location*			
Metropolitan	668 (63.8%)	2156 (77.6%)	< 0.001
Regional	334 (31.9%)	426 (15.3%)	
Interstate/overseas	8 (0.8%)	102 (3.7%)	
Missing	37 (3.5%)	95 (3.4%)	
*IRSAD category*			
1—lowest	123 (11.7%)	236 (8.5%)	< 0.001
2	161 (15.4%)	285 (10.3%)	
3	191 (18.2%)	362 (13.0%)	
4	210 (20.1%)	673 (24.2%)	
5—highest	358 (34.2%)	1216 (43.8%)	
Missing	4 (0.4%)	7 (0.3%)	
Age at surgery, median (IQR)	65.6 (60.3, 69.6)	65.0 (59.6, 69.3)	0.047
Years between diagnosis and surgery, median (IQR)	0.2 (0.1, 0.3)	0.2 (0.1, 0.3)	< 0.001
Years between surgery and follow-up, median (IQR)	1.0 (1.0, 1.1)	1.0 (1.0, 1.1)	0.86
*PSA at surgery (ng/ml)*			
< 4.8	202 (19.3%)	774 (27.9%)	< 0.001
4.81–6.4	240 (22.9%)	661 (23.8%)	
6.41–9	286 (27.3%)	658 (23.7%)	
> 9	298 (28.5%)	632 (22.7%)	
Missing	21 (2.0%)	54 (1.9%)	
*Pathological T score*			
T2	489 (46.7%)	1363 (49.0%)	< 0.001
T3	472 (45.1%)	1362 (49.0%)	
T4	4 (0.4%)	2 (0.1%)	
Missing	82 (7.8%)	52 (1.9%)	
*Surgery Gleason Score*			
6 or less	60 (5.7%)	98 (3.5%)	< 0.001
7	771 (73.6%)	2301 (82.8%)	
8	61 (5.8%)	114 (4.1%)	
9	147 (14.0%)	241 (8.7%)	
10	1 (0.1%)	0	
Missing	7 (0.7%)	25 (0.9%)	
*ISUP grade group at surgery*			
1	136 (13.0%)	357 (12.8%)	< 0.001
2	430 (41.1%)	1261 (45.4%)	
3	306 (29.2%)	835 (30.0%)	
4	80 (7.6%)	147 (5.3%)	
5	91 (8.7%)	156 (5.6%)	
Missing	4 (0.4%)	23 (0.8%)	
*Surgical margins*			
Absent	668 (63.8%)	2175 (78.3%)	< 0.001
Present	356 (34.0%)	566 (20.4%)	
Missing	23 (2.2%)	38 (1.4%)	
*Surgical institution type*			
Public	412 (39.4%)	402 (14.5%)	< 0.001
Private	634 (60.6%)	2370 (85.3%)	
Missing	1 (0.1%)	7 (0.3%)	
*Surgical institution location*			
Metropolitan	749 (71.5%)	2599 (93.5%)	< 0.001
Regional	297 (28.4%)	173 (6.2%)	
Interstate/overseas	0 (0.0%)	3 (0.1%)	
Missing	1 (0.1%)	4 (0.1%)	
*Surgeon year of specialisation*			
1974–1990	139 (13.3%)	633 (22.8%)	< 0.001
1991–2000	430 (41.1%)	312 (11.2%)	
2001–2006	90 (8.6%)	799 (28.8%)	
2007–2009	185 (17.7%)	434 (15.6%)	
2010–2018	156 (14.9%)	568 (20.4%)	
Missing	47 (4.5%)	33 (1.2%)	
*Surgeon years since specialisation*			
0–6	167 (16.0%)	631 (22.7%)	< 0.001
7–10	169 (16.1%)	630 (22.7%)	
11–16	181 (17.3%)	576 (20.7%)	
17–26	339 (32.4%)	321 (11.6%)	
27–44	143 (13.7%)	588 (21.2%)	
Missing	48 (4.6%)	33 (1.2%)	

All continuous variables used a two-sample t-test. All categorical variables used a Pearson’s chi-squared test. *P* values exclude missing data

TRUS = Transrectal ultrasound, TUR* P* = Transurethral resection of the prostate, PSA = Prostate-Specific Antigen, ISU* P* = International Society of Urological Pathology, NCCN = National Comprehensive Cancer Network, IRSAD = Index of Relative Socioeconomic Advantage and Disadvantage

Table 2 shows the covariate balance in the propensity score matched cohort, compared to the unmatched cohort. After propensity score matching, no variables exceeded a standardised difference of 8%.

**Table 2:** Covariate balance table and standardised differences of variables included in the propensity score model

Factor	Unmatched cohort				Matched cohort			
	ORP	RARP	*P* value	Standardised difference	ORP	RARP	*P* value	Standardised difference
N	971	2663			3634	3634		
Age at surgery, mean (SD)	64.9 (6.8)	64.3 (7.1)	0.028	− 8.3%	64.4 (6.9)	64.7 (7.0)	0.100	3.8%
PSA at surgery, mean (SD)	8.4 (6.4)	7.9 (7.4)	0.033	− 8.3%	7.9 (6.1)	8.0 (8.1)	0.510	1.5%
Surgeon years since specialisation, mean (SD)	16.4 (9.3)	15.8 (11.3)	0.120	− 6.0%	16.5 (8.4)	16.4 (11.5)	0.540	− 1.4%
*NCCN category*								
Low risk	85 (8.8%)	240 (9.0%)	< 0.001		325 (8.9%)	321 (8.8%)	0.630	
Intermediate risk	617 (63.5%)	1853 (69.6%)		12.8%	2488 (68.5%)	2479 (68.2%)		− 0.5%
High risk	236 (24.3%)	526 (19.8%)		− 11.0%	733 (20.2%)	760 (20.9%)		1.8%
Metastatic	33 (3.4%)	44 (1.7%)		− 11.1%	88 (2.4%)	74 (2.0%)		− 2.6%
*Hospital location*								
Metropolitan	690 (71.1%)	2493 (93.6%)	< 0.001		3161 (87.0%)	3184 (87.6%)	0.420	
Regional	281 (28.9%)	170 (6.4%)		− 61.9%	473 (13.0%)	450 (12.4%)		− 1.9%
*Hospital type*								
Public	351 (36.1%)	366 (13.7%)	< 0.001		715 (19.7%)	697 (19.2%)	0.590	
Private	620 (63.9%)	2297 (86.3%)		53.6%	2919 (80.3%)	2937 (80.8%)		1.3%
*IRSAD category*								
1—lowest	112 (11.5%)	224 (8.4%)	< 0.001		274 (7.5%)	376 (10.3%)	< 0.001	
2	150 (15.4%)	272 (10.2%)		− 15.7%	395 (10.9%)	391 (10.8%)		− 0.4%
3	175 (18.0%)	345 (13.0%)		− 14.0%	515 (14.2%)	528 (14.5%)		1.0%
4	195 (20.1%)	652 (24.5%)		10.6%	937 (25.8%)	819 (22.5%)		− 7.6%
5—highest	339 (34.9%)	1170 (43.9%)		18.5%	1513 (41.6%)	1520 (41.8%)		0.4%
*Year of surgery*								
2014	153 (15.8%)	417 (15.7%)	0.230		467 (12.9%)	552 (15.2%)	0.040	
2015	194 (20.0%)	598 (22.5%)		6.1%	759 (20.9%)	763 (21.0%)		0.3%
2016	275 (28.3%)	709 (26.6%)		− 3.8%	995 (27.4%)	960 (26.4%)		− 2.2%
2017	287 (29.6%)	807 (30.3%)		1.6%	1193 (32.8%)	1126 (31.0%)		− 4.0%
2018	62 (6.4%)	132 (5.0%)		− 6.2%	220 (6.1%)	233 (6.4%)		1.5%

Based on the propensity score matching model, the matching process created an observation for each patient if they were to receive the other treatment option and imputed the potential outcome of each observation. Therefore the matched cohort contained double the amount of observations (n = 7268) than the unmatched cohort (n = 3634). Before propensity score matching, standardised differences between ORP and RARP groups exceeded 10% for four of nine analysed covariates (NCCN category, hospital type, hospital location and IRSAD quintile). After propensity score matching, no variables exceeded a standardised difference of 8%

All continuous variables used a two-sample t-test. All binary and categorical variables used the Pearson’s chi-squared test. The matched cohort shows the distribution of covariates after matching for all patients that had answered the urinary bother item, as it had the highest number of responses out of all outcome variables

### Urinary bother

In the unmatched cohort, there was no statistically significant difference in the risk of reporting moderate/big urinary bother in RARP compared to ORP patients (Rd = − 1.69%, *P* = 0.125) (Table 3)*.* After adjusting for baseline characteristics in the propensity score matched cohort, there was also no significant difference in reporting moderate/big urinary bother (Rd = 0.47%, *P* = 0.707) (Table 3).

**Table 3:** Propensity score matching results for EPIC-26 items

Binary outcomes	Unmatched cohort						Matched cohort					
	ORP	RARP	Difference	*P* value	95% CI		ORP	RARP	Difference	*P* value	95% CI	
*Urinary outcomes*												
Urinary bother (moderate-big)	9.99%	8.30%	− 1.69%	0.125	− 3.85%	0.47%	7.93%	8.39%	0.47%	0.707	− 1.97%	2.91%
Pad usage (≥1 pad)	33.53%	30.23%	− 3.36%	0.074	− 7.03%	0.32%	31.48%	30.73%	− 0.75%	0.771	− 5.76%	4.27%
Urinary incontinence domain, mean (SD)	76.98 (25.64)	78.25 (24.00)	1.27	0.195	− 3.19	0.65	78.82 (24.31)	77.99 (23.93)	− 0.84	0.506	− 3.30	1.63
Urinary irritative/obstructive domain, mean (SD)	92.48 (11.59)	93.47 (10.56)	1.00	0.022	− 1.85	− 0.14	92.48 (11.35)	93.50 (10.64)	1.03	0.105	− 0.21	2.27
*Sexual outcomes*												
Sexual bother (moderate-big)	39.73%	39.40%	− 0.33%	0.860	− 3.95%	3.30%	39.38%	38.49%	− 0.89%	0.731	− 5.97%	4.19%
Sexual domain, mean (SD)	23.48 (22.24)	30.15 (24.56)	6.67	<0.001	− 8.56	− 4.78	25.92 (24.24)	29.57 (24.35)	3.65	0.005	1.10	6.20

This table compares binary outcome variables (urinary bother, sexual bother and pad usage) and continuous outcome variables (urinary incontinence domain, urinary irritative/obstructive domain and sexual domain) between men who underwent ORP and RARP in both unmatched and matched cohorts. For the binary variables, the unmatched cohort used binary regression, expressed as a risk difference (RARP-ORP), whereas the matched cohort used propensity score matching average treatment effects, expressed as a risk difference (RARP-ORP). For continuous variables, the unmatched cohort used t-tests expressed as a mean difference, which is the difference in mean scores between groups (RARP-ORP), whereas the matched cohort used propensity score matching average treatment effects expressed as a mean difference

Unmatched cohort: Urinary bother population (n = 3634): OR*P* = 971, RARP = 2663. Pad usage population (n = 3215): ORP = 850, RARP = 2365. Urinary incontinence population (n = 3203): ORP = 845, RARP = 2358. Urinary irritative/obstructive population (n = 3198): ORP = 845, RARP = 2353. Sexual bother population (n = 3596): ORP = 954, RARP = 2642. Sexual domain population (n = 3179): ORP = 838 RARP = 2341

ORP = Open radical prostatectomy; RARP = Robot-assisted radical prostatectomy; Difference = risk difference (binary variables) or mean difference (continuous variables); 95% CI = 95% confidence interval

### Urinary incontinence

In the unmatched cohort, there was no significant difference in urinary incontinence domain scores in RARP patients compared to ORP patients (Coeff = 1.27, *P* = 0.195). In the propensity score matched cohort, there was also no significant difference in scores (Coeff = − 0.84, *P* = 0.506).

### Pad usage

In the unmatched cohort, there was no significant difference in the risk of wearing ≥ 1 pad per day in RARP patients (Rd = − 3.36%, *P* = 0.074). In the propensity score matched cohort, there remained no significant difference in wearing ≥ 1 pad per day (Rd = 0.75%, *P* = 0.771). Sensitivity analysis with different cut-offs for binary variables can be seen in Additional file 1: Table S1, showing no difference between ORP and RARP in terms of pad usage, urinary bother and sexual bother.

### Sexual bother

In the unmatched cohort, there was no significant difference in the risk of reporting moderate/big sexual bother in RARP compared to ORP patients (Rd = − 0.33%, *P* = 0.860). In the propensity score matched cohort, the difference remained insignificant (Rd = − 0.89%, *P* = 0.731).

### Sexual domain

In the unmatched sample, there were superior outcomes for men undergoing RARP, compared to those undergoing ORP, in sexual domain score (30.15 vs 23.48, < 0.001. In the propensity score matched cohort, this superiority persisted, but was less pronounced (29.57 vs. 25.92, respectively, *P* = 0.005).

## Discussion

This study used a large, registry-based cohort of patients and evaluated one-year urinary and sexual PROs. The unmatched cohort had different baseline characteristics, which we propose is due to a disparity in access to RARP. RARP patients were more likely to undergo surgery at metropolitan hospitals than ORP patients (93.5% vs. 71.5%). RARP patients were also more likely to undergo surgery at private hospitals than ORP patients (85.3% vs. 60.6%). RARP patients had lower risk disease, which is potentially due to earlier screening.

Propensity score matching was used to decrease these and other baseline differences. After propensity score matching, no variables exceeded a standardised difference of 8%. Many covariates had major reductions in standardised difference, including hospital location, hospital type and IRSAD category. The differences in four out of six PROs decreased once patient and surgeon characteristics were more even amongst matched groups. This may be slightly due to an increased sample size from matching but is more likely due to a reduction in baseline differences.

Our results show that there is no statistically significant difference between ORP and RARP in reporting any urinary outcomes, including urinary bother, urinary incontinence, urinary irritative/obstructive and pad usage. Furthermore, there was no statistically significant difference between ORP and RARP in reporting sexual bother. Sexual domain scores were statistically superior in the RARP group. However, an absolute difference of less than 4 points on a 100-point scale was deemed unlikely to be clinically significant [23]. In large datasets, including those collected by clinical registries, small differences in outcomes between treatment groups may be considered statistically significant even though they are not clinically meaningful [24].

### Urinary outcomes

The largest RCT in the field reported no difference in urinary incontinence domain scores at 6 weeks, 12 weeks and 6-, 12- and 24-months [6, 7]. However, they only used two surgeons with varying levels of experience and therefore their findings may be confounded by surgeon differences. Three studies have used propensity score matching to compare postoperative urinary outcomes between ORP and RARP [18, 25, 26]. Each reported no difference in urinary outcomes between ORP and RARP (Additional file 1: Table S1) [18, 25, 26].

The majority of studies in the field are observational and have retrospectively compared ORP and RARP. A previous study from the PCOR-Vic found no difference in 12-month urinary bother [18]. In contrast, Herlemann et al. concluded that men undergoing ORP were more likely to report superior EPIC-26 urinary incontinence domain scores and less bother than RARP patients within one year of surgery, in unadjusted analysis [27]. However, they also noted that the ORP group had significantly lower risk scores, Gleason grades and pT stages, which introduced selection bias. Results like these should be adjusted for baseline differences through techniques such as propensity score matching or regression analysis. Herlemann et al. hypothesised that the worse urinary outcomes for RARP patients in the first year may be influenced by increased expectations of RARP, especially in America [27].

One Swedish study found no difference between ORP and RARP in the number of pads worn per day for all daily cut-offs (≥ 1, ≥ 2, ≥ 4 and ≥ 6) [28]. We also found no difference between ORP and RARP patients in the unmatched and matched cohorts, with cut-offs at ≥ 1, ≥ 2 and ≥ 3 pads per day (Additional file 1: Table S2).

### Sexual outcomes

Our finding of marginal improvement in sexual domain scores at 12 months in the RARP group is comparable with two observational studies that used propensity score matching [25, 29]. An Italian study by Antonelli et al. found a significant difference in 6-month unadjusted sexual function scores in favour of RARP by 12.41 points (*p* < 0.001), using the University of California Los Angeles Prostate Cancer Index (UCLA-PCI) questionnaire [25]. However, there was no difference at 12 months [25]. Antonelli et al. noted a clear trend towards centralisation of RARPs in Italy and therefore surgeon factors and volume-outcome relationships might explain the superior outcomes for RARP [25]. Similarly, RARP is centralised to metropolitan regions in Victoria, with greater access in private hospitals, which may skew views of its superiority.

Sooriakumaran et al. reported that patients in low or intermediaterisk groups recovered earlier from RARP, based on a single 5-scale penile stiffness question [29]. This was claimed to be due to RARP being a more flexible technique that has a greater capacity for nerve-sparing [29].

In a large American cohort, O’Neil et al. found a strong relationship between baseline function and function at later timepoints using common items from the UCLA-PCI and EPIC questionnaires to form a modified sexual domain summary score [30]. Above a baseline sexual function score of 62/100, RARP patients were found to have significantly improved sexual function at 12 months compared to ORP patients by a magnitude of 2.70 to 10.31 points [30]. This may indicate that RARP is able to preserve function in patients with higher preoperative function.

### Strengths and limitations

Our study included data from 100 surgeons across metropolitan and regional Victoria. The use of propensity score matching allowed us to mimic a RCT by using matching in place of randomisation to create ORP and RARP patients who were alike on important baseline factors. It is important to understand the intricacies of each adjustment model and the variables selected in each model to assess the significance of outcomes. In contrast to other propensity score matching studies that used inverse probability treatment weighting [18, 25, 26, 29] we used 1:1 nearest neighbour matching, with replacement and no calipers. We included a number of known and novel factors in our propensity score model. Given the evidence that surgeon experience and skill are undervalued determinants of patient outcomes [31], our model included a novel variable, the number of years since the surgeon’s specialisation. Furthermore, hospital location (metropolitan vs. regional) was included, which has not yet been examined as a predictor of quality of life. The effect of hospital type (private vs. public) will vary depending on each country’s healthcare system. However, we included a hospital type variable as Victorian private sector patients have an increased likelihood of receiving RARP than public sector patients [32].

This study is not without limitations. First, the observational study design introduces the potential of unmeasured confounding and bias. The analysis was limited to outcome data collected at one timepoint and therefore no inferences could be made regarding time to recovery. Second, the absence of PRO data at baseline prevents adjusting for preoperative function. Participants of the PCOR-Vic are identified and contacted for consent a few months following diagnosis. Therefore collection of baseline data is not possible as many men will have had active management by the time they enter the registry. Furthermore, other variables were not collected by PCOR-Vic and therefore not included in the analysis, such as previous urological surgery and comorbidity data. Third, surgeries in the public sector are often performed by *trainees* under the supervision of *nominal* surgeons, and surgeon learning curve factors were not assessed. Therefore, our stratification of surgeon experience may not be truly representative. Fourth, the PCOR-Vic database had 75% coverage of all RPs in Victoria in 2013 [33] and 89% population coverage in 2019 [34], and captured data from patients at 61 sites (30/33 public and 31/41 private). We believe the available data is representative of the Victorian population, however may not be applicable to other settings. Fifth, the PCOR-Vic database lacks standardised data collection for specimen handling and histological subtyping across all sites.

We suggest that future studies should capture surgeon-specific factors such as experience, skill and technique as well as ancillary services offered in the public and private sectors. Assessment of centralising services to high-volume centres is required as it may pool resources to better manage needs, yet may increase disparities in access to care [2]. Furthermore, the emergence of new surgical and radiological techniques, adjuvant treatments and medications, and demographic changes in patients and surgeons has the potential to change outcomes following RP and therefore requires ongoing assessment.

## Conclusion

In this state-wide cohort of patients receiving ORP or RARP, there were no clinically significant differences in urinary PROs at one-year following RP. In terms of sexual function, there was slightly superior sexual domain scores for the RARP group, which was not deemed clinically significant.

## Supplementary Information

**Additional file 1**. **Figure S1.** Balance plot of the probability of receiving RARP in both the ORP and RARP groups, in the unmatched and propensity score matched cohorts. **Table S1.** Summary of propensity score-matched studies comparing urinary and/or sexual outcomes following ORP vs. RARP. **Table S2.** Sensitivity analysis of binary outcomes dichotomised into different groups also found no significant differences between ORP and RARP in the unmatched or matched cohorts. **Table S3.** 12-month EPIC-26 urinary incontinence domain full responses. All items in the EPIC-26 questionnaire refer to the last 4 weeks experienced by patients. This table compares individual items from the urinary incontinence domain between ORP and RARP. **Table S4.** 12-month EPIC-26 urinary irritative/obstructive domain full responses. **Table S5.** 12-month EPIC-26 sexual domain full responses. **Table S6.** Other management/treatment options in cohort. **Table S7.** Subgroup analysis of the use of medications and aids for erectile function.

## Data Availability

Raw data were generated at the Prostate Cancer Outcomes Registry—Victoria. Derived data supporting the findings of this study are available from the corresponding author on request.

## Abbreviations

RARP: Robot-assisted radical prostatectomy
ORP: Open radical prostatectomy
PRO: Patient-reported outcome
RP: Radical prostatectomy
EPIC: Expanded Prostate cancer Index Composite questionnaire
RCT: Randomised controlled trial
IRSAD: Index of relative socioeconomic advantage and disadvantage
