# Supplementary material for: Comparison of urinary and sexual patient-reported outcomes between open radical prostatectomy and robot-assisted radical prostatectomy: a propensity score matched, population-based study in Victoria
Source: BMC Urol. 2022 Feb 7;22:18. doi: 10.1186/s12894-022-00966-0 (PMC8822814; doi:10.1186/s12894-022-00966-0)
Supplement: Supplementary file 1 — Additional file 1. Figure S1. Balance plot of the probability of receiving RARP in both the ORP and RARP groups, in the unmatched and propensity score matched cohorts. Table S1. Summary of propensity score-matched studies comparing urinary and/or sexual outcomes following ORP vs. RARP. Table S2. Sensitivity analysis of binary outcomes dichotomised into different groups also found no significant differences between ORP and RARP in the unmatched or matched cohorts. Table S3. 12-month EPIC-26 urinary incontinence domain full responses. All items in the EPIC-26 questionnaire refer to the last 4 weeks experienced by patients. This table compares individual items from the urinary incontinence domain between ORP and RARP. Table S4. 12-month EPIC-26 urinary irritative/obstructive domain full responses. Table S5. 12-month EPIC-26 sexual domain full responses. Table S6. Other management/treatment options in cohort. Table S7. Subgroup analysis of the use of medications and aids for erectile function. [file 12894_2022_966_MOESM1_ESM.docx]

**Additional File 1 (Supplementary material)**

**Figure S1.** **Balance plot of the probability of receiving RARP in both the ORP and RARP groups, in the unmatched and propensity score matched cohorts.** After matching, the treatments groups become more similar in the distribution of probabilities of receiving RARP, as seen through the overlapping of probability distribution density. This is due to the groups having greater similarity in the baseline characteristics included in the model.

**Table S1.** **Summary of propensity score-matched studies comparing urinary and/or sexual outcomes following ORP vs. RARP.**

| **Author (year)** | **Registry/trial/hospital, nation** | **Timespan of surgery** | **Main outcome of interest timepoint** | **No. of patients in cohort** | **No. of patients in propensity score cohort** | **Variables included in propensity score model** | **Conditions of propensity score model** | **PROM assessment tools** | **Main urinary function endpoint** | **Main urinary function findings** | **Main sexual function endpoint** | **Main sexual function findings** |
| --- | --- | --- | --- | --- | --- | --- | --- | --- | --- | --- | --- | --- |
| Antonelli et al (2019)^+^ | Pros-IT CNR, Italy | 2014-2015 | 6, 12 mo | ORP (115) vs. LRP (90) vs. RARP (336) | ORP (115) vs. LRP (90) vs. RARP (336) | Age, education, diabetes, comorbidities, family history of PCa, biopsy Gleason score, Several baseline HRQoL variables | IPTW | Italian UCLA-PCI | UB;  UF | No difference | SB;  SF | Superior sexual function at 6 mo in RARP;  Superior erections firm enough for intercourse at 6 & 12 mo in RARP |
| Koo et al (2014) | Severance Hospital cohort, Korea | 1992-2008 | 12 mo | ORP (580) vs. RARP (592) | ORP (175) vs. RARP (175) | Patient age, preoperative PSA, biopsy Gleason score, cT stage | N/S | N/S | Pad use* | No difference | N/A | N/A |
| Ong et al (2016) | PCOR-Vic registry, Australia | 2009-2013 | 12 mo | ORP (1117) vs. RARP (885) | N/S | Age, PSA level, NCCN, hospitals, year of RP, interval between RP and HRQOL outcomes assessment, and average annual caseload | IPTW | EPIC-26 | Urinary bother question | No difference | SB | No difference |
| Sooriakumaran et al (2018) | LAPPRO trial, Sweden | 2008-2011 | 3, 12, 24 mo | ORP (753) vs. RARP (1792) | N/S | Age at surgery, educational level, smoking, employment, CVD, cT stage, biopsy Gleason score, PSA, total mm cancer in biopsy cores | IPTW | N/S | N/A | N/A | Penile stiffness question  Morning erection question | Earlier recovery for low-risk patients in RARP  Less erectile problems for high-risk patients in ORP |
| Present study | Australia, PCOR-Vic registry | 2014-2018 | 12 mo | ORP (1047) vs. RARP (2779) | ORP (3826) vs. RARP (3826) | Age at surgery, surgery year, surgery PSA level, NCCN risk category, Metro vs. regional surgical institution, public vs. private surgical institution, IRSAD quintile, surgeon years since specialisation | Propensity score matching | EPIC-26 | UB, urinary incontinence domain, pad use | No difference | SB, sexual function domain | No difference |
| CaPSURE = Cancer of the Prostate Strategic Urologic Research Endeavor  Pros-IT CNR = Prostate-Italy National Research Council project  LAPPRO = LAParoscopic Prostatectomy Robot Open  PCOR-Vic = Prostate Cancer Outcomes Registry – Victoria  + = Study included LRP | | | | UCLA-PCI = University of California Los Angeles-prostate cancer index  IIEF = International Index of Erectile Function  EPIC = Expanded Prostate Index Composite score  IPTW = Inverse Probability Treatment Weighting  UB = Urinary bother; SB = Sexual bother; UF= Urinary Function; SF= Sexual Function;  HRQOL = Health-related Quality of Life | | | | ^Good function defined as “at least once per 24 hours”  *Good function defined as “absence of pads”  $= Good function defined as “most times” or “almost always or always.”  N/A = Not Applicable  N/S = Not Stated  mo = months | | | | |

**Table S2. Sensitivity analysis of binary outcomes dichotomised into different groups also found no significant differences between ORP and RARP in the unmatched or matched cohorts.**

| **Binary outcomes** | **Unmatched cohort** | | | | | | **Matched cohort** | | | | | |
| --- | --- | --- | --- | --- | --- | --- | --- | --- | --- | --- | --- | --- |
|  | **ORP** | **RARP** | **Rd** | **P-value** | **95% CI** | | **ORP** | **RARP** | **Rd** | **P-value** | **95% CI** | |
| Urinary Bother (big bother) | 2.68% | 2.59% | -0.09% | 0.886 | -1.27% | 1.09% | 2.56% | 2.37% | -0.19% | 0.802 | -1.70% | 1.31% |
| Sexual Bother (big bother) | 30.82% | 28.24% | -2.58% | 0.136 | -5.98% | 33.75% | 29.09% | 27.50% | -1.59% | 0.513 | -6.33% | 3.16% |
| Pad usage (≥2 pad) | 8.12% | 6.60% | -1.52% | 0.154 | -3.61% | 0.57% | 6.78% | 6.69% | -0.19% | 0.875 | -2.52% | 2.14% |
| Pad usage (≥3 pad) | 3.18% | 3.00% | -0.17% | 0.802 | -1.54% | 1.19% | 3.64% | 2.86% | -0.78% | 0.399 | -2.59% | 1.03% |
| ORP = Open radical prostatectomy; RARP = Robot-assisted radical prostatectomy; Rd= Risk difference; 95% CI = 95% confidence interval  The unmatched cohort used binary regression, expressed as a risk difference (RARP-ORP). The matched cohort used propensity score matching average treatment effect, expressed as a risk difference (RARP-ORP)  Urinary bother population: (n=3634) ORP = 971, RARP=2663  Sexual bother population: (n=3596) ORP=954, RARP=2642  Pad usage population: (n=3215) ORP=850, RARP=2365 | | | | | | | | | | | | |

**Table S3. 12-month EPIC-26 urinary incontinence domain full responses.** All items in the EPIC-26 questionnaire refer to the last 4 weeks experienced by patients. This table compares individual items from the urinary incontinence domain between ORP and RARP.

| **Factor** | **ORP** | **RARP** | **P-value** |
| --- | --- | --- | --- |
| N | 1047 | 2779 |  |
| How often have you **leaked** urine? |  |  | 0.007 |
| More than once a day | 197 (21.5%) | 427 (17.3%) |  |
| About once a day | 108 (11.8%) | 312 (12.7%) |  |
| More than once a week | 58 (6.3%) | 190 (7.7%) |  |
| About once a week | 102 (11.1%) | 355 (14.4%) |  |
| Rarely or never | 453 (49.3%) | 1181 (47.9%) |  |
| Which of the following best describes your **urinary control**? |  |  | 0.06 |
| No urinary control whatsoever | 13 (1.4%) | 25 (1.0%) |  |
| Frequent dribbling | 71 (7.7%) | 134 (5.4%) |  |
| Occasional dribbling | 397 (43.2%) | 1083 (43.9%) |  |
| Total control | 437 (47.6%) | 1223 (49.6%) |  |
| How many **pads** or adult diapers per day did you usually use to control leakage? |  | | 0.11 |
| 3 or more pads per day | 33 (3.6%) | 77 (3.1%) |  |
| 2 pads per day | 44 (4.8%) | 89 (3.6%) |  |
| 1 pad per day | 238 (25.9%) | 579 (23.5%) |  |
| None | 604 (65.7%) | 1721 (69.8%) |  |
| How big a problem, if any, has **dripping or leaking** urine been? |  |  | 0.065 |
| Big problem | 43 (4.7%) | 75 (3.0%) |  |
| Moderate problem | 60 (6.5%) | 136 (5.5%) |  |
| Small problem | 127 (13.8%) | 328 (13.3%) |  |
| Very small problem | 215 (23.4%) | 651 (26.4%) |  |
| No problem | 473 (51.5%) | 1275 (51.7%) |  |

**Table S4.** **12-month EPIC-26 urinary irritative/obstructive domain full responses**

| **Factor** | **ORP** | **RARP** | **p-value** |
| --- | --- | --- | --- |
| N | 1047 | 2779 |  |
| Pain or burning on urination |  |  | 0.84 |
| Big problem | 4 (0.4%) | 7 (0.3%) |  |
| Moderate problem | 7 (0.8%) | 17 (0.7%) |  |
| Small problem | 13 (1.4%) | 26 (1.1%) |  |
| Very small problem | 24 (2.6%) | 59 (2.4%) |  |
| No problem | 870 (94.8%) | 2353 (95.6%) |  |
| Bleeding with urination |  |  | 0.12 |
| Big problem | 1 (0.1%) | 2 (0.1%) |  |
| Moderate problem | 2 (0.2%) | 0 (0.0%) |  |
| Small problem | 4 (0.4%) | 5 (0.2%) |  |
| Very small problem | 5 (0.5%) | 9 (0.4%) |  |
| No problem | 907 (98.7%) | 2445 (99.3%) |  |
| Weak urine stream or incomplete emptying |  |  | <0.001 |
| Big problem | 10 (1.1%) | 19 (0.8%) |  |
| Moderate problem | 36 (3.9%) | 75 (3.1%) |  |
| Small problem | 74 (8.1%) | 109 (4.4%) |  |
| Very small problem | 103 (11.2%) | 242 (9.8%) |  |
| No problem | 693 (75.7%) | 2012 (81.9%) |  |
| Need to urinate frequently during the day |  |  | 0.16 |
| Big problem | 22 (2.4%) | 44 (1.8%) |  |
| Moderate problem | 75 (8.2%) | 155 (6.3%) |  |
| Small problem | 89 (9.7%) | 278 (11.3%) |  |
| Very small problem | 157 (17.1%) | 412 (16.7%) |  |
| No problem | 575 (62.6%) | 1573 (63.9%) |  |

**Table S5.** **12-month EPIC-26 sexual domain full responses**

| **Factor** | **ORP** | **RARP** | **p-value** |
| --- | --- | --- | --- |
| N | 1047 | 2779 |  |
| How would you rate your **ability to have an erection**? |  |  | <0.001 |
| Very poor to none | 640 (70.3%) | 1498 (61.0%) |  |
| Poor | 120 (13.2%) | 355 (14.5%) |  |
| Fair | 90 (9.9%) | 313 (12.8%) |  |
| Good | 41 (4.5%) | 209 (8.5%) |  |
| Very good | 19 (2.1%) | 79 (3.2%) |  |
| How would you rate your **ability to reach orgasm (climax)**? |  |  | <0.001 |
| Very poor to none | 474 (52.7%) | 1011 (41.6%) |  |
| Poor | 110 (12.2%) | 292 (12.0%) |  |
| Fair | 130 (14.5%) | 428 (17.6%) |  |
| Good | 127 (14.1%) | 475 (19.6%) |  |
| Very good | 58 (6.5%) | 222 (9.1%) |  |
| How would you describe the usual **QUALITY** of your erections? |  |  | <0.001 |
| None at all | 556 (61.1%) | 1214 (49.6%) |  |
| Not firm enough for any sexual activity | 147 (16.2%) | 406 (16.6%) |  |
| Firm enough for masturbation and foreplay only | 123 (13.5%) | 454 (18.6%) |  |
| Firm enough for intercourse | 84 (9.2%) | 372 (15.2%) |  |
| How would you describe the **FREQUENCY** of your erections? |  |  | <0.001 |
| Never | 682 (75.4%) | 1590 (65.2%) |  |
| Less than half the time | 70 (7.7%) | 220 (9.0%) |  |
| About half the time | 53 (5.9%) | 192 (7.9%) |  |
| More than half the time | 38 (4.2%) | 171 (7.0%) |  |
| Whenever I wanted one | 62 (6.9%) | 265 (10.9%) |  |
| Overall, how would you rate your **ability to function sexually**? |  |  | <0.001 |
| Very poor | 635 (70.0%) | 1418 (58.1%) |  |
| Poor | 112 (12.3%) | 351 (14.4%) |  |
| Fair | 92 (10.1%) | 338 (13.8%) |  |
| Good | 49 (5.4%) | 248 (10.2%) |  |
| Very good | 19 (2.1%) | 86 (3.5%) |  |
| Overall, how big a **problem** has your sexual function or lack of sexual function been for you? |  |  | 0.004 |
| Big bother | 321 (31.2%) | 771 (28.1%) |  |
| Moderate bother | 87 (8.5%) | 304 (11.1%) |  |
| Small bother | 165 (16.1%) | 459 (16.7%) |  |
| Very small bother | 181 (17.6%) | 574 (20.9%) |  |
| No bother | 274 (26.7%) | 640 (23.3%) |  |

**Table S6. Other management/treatment options in cohort**

| **Factor** | **ORP** | **RARP** | **P-value** |
| --- | --- | --- | --- |
| N | 1047 | 2779 |  |
| Active Surveillance |  |  |  |
| No | 974 (93.0%) | 2594 (93.3%) | 0.73 |
| Yes | 73 (7.0%) | 185 (6.7%) |  |
| Radiotherapy (any) |  |  |  |
| No | 943 (90.1%) | 2653 (95.5%) | <0.001 |
| Yes | 104 (9.9%) | 126 (4.5%) |  |
| Postoperative RT (within 1 year following RP) |  |  |  |
| No | 950 (90.7%) | 2686 (96.7%) | <0.001 |
| Yes | 97 (9.3%) | 93 (3.3%) |  |
| ADT |  |  |  |
| No | 1024 (97.8%) | 2723 (98.0%) | 0.72 |
| Yes | 23 (2.2%) | 56 (2.0%) |  |
| Chemotherapy |  |  |  |
| No | 1041 (99.4%) | 2754 (99.1%) | 0.32 |
| Yes | 6 (0.6%) | 25 (0.9%) |  |

**Table S7. Subgroup analysis of the use of medications and aids for erectile function.** These data were only collected from December 2016 and therefore was only included in subgroup analysis. Chi-squared tests was used for this categorical data. RARP patients were significantly more likely to report being more interested in sex (P=0.008), using any erectile medication/aid (P<0.001), using Viagra or another pill for erectile function (P<0.001) and using a vacuum erection device (P=0.009).

|  |  |  |  |
| --- | --- | --- | --- |
| **Factor** | **ORP** | **RARP** | **P-value** |
| N | 694 | 1764 |  |
| 1. During the last 4 weeks, to what extent were you interested in sex? |  |  | 0.008 |
| Declined to answer | 11 (1.6%) | 14 (0.8%) |  |
| Not at all | 180 (26.0%) | 357 (20.3%) |  |
| A little | 254 (36.7%) | 679 (38.7%) |  |
| Quite a bit | 187 (27.0%) | 549 (31.3%) |  |
| Very much | 61 (8.8%) | 156 (8.9%) |  |
| 2. Have you used any medications or devices to aid or improve erections? |  |  | <0.001 |
| Declined to answer | 6 (0.9%) | 8 (0.5%) |  |
| No | 378 (54.9%) | 814 (46.6%) |  |
| Yes | 305 (44.3%) | 923 (52.9%) |  |
| 3. Please indicate whether or not you have tried or currently use: |  |  |  |
|  |  |  |  |
| 3A. Viagra or another pill to improve your erections? |  |  | <0.001 |
| Declined to answer | 6 (0.9%) | 12 (0.7%) |  |
| Have not tried it | 380 (55.0%) | 790 (44.9%) |  |
| Tried it but was not helpful | 202 (29.2%) | 561 (31.9%) |  |
| It helped but I am not using it now | 25 (3.6%) | 91 (5.2%) |  |
| It helped and I use it sometimes | 46 (6.7%) | 192 (10.9%) |  |
| It helped and I use it always | 32 (4.6%) | 113 (6.4%) |  |
| 3B. Muse (intra‐urethral alprostadil suppository) to improve your erections? |  |  | 0.34 |
| Declined to answer | 6 (0.9%) | 8 (0.5%) |  |
| Have not tried it | 680 (98.6%) | 1734 (99.3%) |  |
| Tried it but was not helpful | 3 (0.4%) | 3 (0.2%) |  |
| It helped and I use it sometimes | 1 (0.1%) | 1 (0.1%) |  |

| **Factor** | **ORP** | **RARP** | **P-value** |
| --- | --- | --- | --- |
| 3C. Penile injection therapy (such as caverject) to improve your erections? |  |  | 0.62 |
| Declined to answer | 6 (0.9%) | 9 (0.5%) |  |
| Have not tried it | 587 (85.1%) | 1465 (83.4%) |  |
| Tried it but was not helpful | 21 (3.0%) | 68 (3.9%) |  |
| It helped but I am not using it now | 29 (4.2%) | 74 (4.2%) |  |
| It helped and I use it sometimes | 32 (4.6%) | 87 (5.0%) |  |
| It helped and I use it always | 15 (2.2%) | 53 (3.0%) |  |
| 3D. Vacuum erection device (such as erect-aid) to improve your erections? |  |  | 0.009 |
| Declined to answer | 6 (0.9%) | 8 (0.5%) |  |
| Have not tried it | 629 (91.2%) | 1523 (86.9%) |  |
| Tried it but was not helpful | 35 (5.1%) | 109 (6.2%) |  |
| It helped but I am not using it now | 7 (1.0%) | 34 (1.9%) |  |
| It helped and I use it sometimes | 10 (1.4%) | 54 (3.1%) |  |
| It helped and I use it always | 3 (0.4%) | 25 (1.4%) |  |
| 3E. Medication/device other than those explicitly listed to improve your erections? |  |  | 0.62 |
| Declined to answer | 6 (0.9%) | 9 (0.5%) |  |
| Have not tried it | 670 (97.0%) | 1695 (96.5%) |  |
| Tried it but was not helpful | 7 (1.0%) | 25 (1.4%) |  |
| It helped but I am not using it now | 4 (0.6%) | 8 (0.5%) |  |
| It helped and I use it sometimes | 2 (0.3%) | 8 (0.5%) |  |
| It helped and I use it always | 2 (0.3%) | 12 (0.7%) |  |
